# Off the Beaten Path in Oncology: Active Brown Adipose Tissue by Virtue of Molecular Imaging

**DOI:** 10.3390/cimb45100499

**Published:** 2023-09-28

**Authors:** Wael Jalloul, Mihaela Moscalu, Roxana Moscalu, Despina Jalloul, Irena Cristina Grierosu, Teodor Ionescu, Cati Raluca Stolniceanu, Vlad Ghizdovat, Veronica Mocanu, Radu Iliescu, Ioana Pavaleanu, Cipriana Stefanescu

**Affiliations:** 1 Department of Biophysics and Medical Physics-Nuclear Medicine, “Grigore T. Popa” University of Medicine and Pharmacy, 700115 Iasi, Romania; jalloul.wael@umfiasi.ro (W.J.); mg-rom-31065@students.umfiasi.ro (D.J.); irena.raileanu@umfiasi.ro (I.C.G.); raluca.stolniceanu@umfiasi.ro (C.R.S.); vlad.ghizdovat@umfiasi.ro (V.G.); cipriana.stefanescu@umfiasi.ro (C.S.); 2 Department of Preventive Medicine and Interdisciplinarity, “Grigore T. Popa” University of Medicine and Pharmacy, 700115 Iasi, Romania; 3 Manchester Academic Health Science Centre, Cell Matrix Biology and Regenerative Medicine, The University of Manchester, Manchester M13 9PT, UK; roxana.moscalu@mft.nhs.uk; 4 Department of Morpho-Functional Sciences (Pathophysiology), “Grigore T. Popa” University of Medicine and Pharmacy, 700115 Iasi, Romania; teodor-marian.ionescu@umfiasi.ro (T.I.); veronica.mocanu@umfiasi.ro (V.M.); 5 Department of Pharmacology, “Grigore T. Popa” University of Medicine and Pharmacy, 700115 Iasi, Romania; radu.iliescu@umfiasi.ro; 6 Department of Mother and Child, “Grigore T. Popa” University of Medicine and Pharmacy, 700115 Iasi, Romania; ioana-m-pavaleanu@umfiasi.ro; 7 North East Regional Innovative Cluster for Structural and Molecular Imaging (Imago-Mol), 700115 Iasi, Romania

**Keywords:** brown adipose tissue, ^18^F-FDG PET/CT, cancer, Cancer-induced Cachexia, BMI

## Abstract

Brown Adipose Tissue (BAT) is considered beneficial in diabetes and obesity, but it can also have negative effects such as its implication in tumours’ pathogenesis and the development of Cancer-induced Cachexia. Since ^18^F-FDG PET/CT is a common molecular imaging modality used in cancer assessment, we aim to study the ^18^F-FDG BAT biodistribution in oncological patients and look for possible correlations between BAT activity and different malignancies as well as the patient’s weight status. After analysing the total number of oncological ^18^F-FDG PET/CT scans between 2017 and 2021, we selected patients with active BAT. Based on their BMI, the selected patients were divided into nonobese (NO) vs. overweight and obese (OOB). OOB SUV_max_lean body mass(LBM) had the highest mean values in supraclavicular, latero-cervical, and paravertebral vs. mediastinal and latero-thoracic localisations in NO. BMI was positively correlated with latero-cervical and supraclavicular SUV_max_(LBM) but negatively correlated with latero-thoracic and abdominal SUV_max_(LBM). Considering the age of the patients, SUV_max_(LBM) decreases in the latero-cervical, paravertebral, and abdominal regions. In addition, the males presented lower SUV_max_(LBM) values. SUV_max_(LBM) was not affected by the treatment strategy or the oncological diagnosis. To conclude, it is mandatory to take into consideration the BAT particularities and effects on weight status in order to optimise the clinical management of oncological patients.

## 1. Introduction

Adipose tissue is divided into three basic types including white adipose tissue (WAT), beige or brite (BeigeAT), and BAT [1]. The main features that differentiate BAT from WAT are represented by smaller and multilocular lipid droplets, different anatomical distributions, greater noradrenergic sympathetic innervation, and the capacity to generate heat. Brown fat also has a high concentration of mitochondria with a dense inner membrane expression of the uncoupling protein 1 (UCP1) [2,3].

Recent research has generated considerable attention regarding the inducible “browning” of white fat [3]. This phenomenon is illustrated by the development of a special adipose cell type known as “brite” fat (“beige fat” or “induced WAT”), which is derived from a particular population of WAT that expresses UCP1 in its mitochondria. In contrast, the classical BAT arises from a mesodermal population distinguished by the expression of muscle lineage markers Myf5+ and Pax7+ [3]. Although they have different developmental origins, BAT and BeigeAT are known to perform similar functions.

As a reaction to acute cold induction, which represents the main factor of its activation [4], BAT produces heat using its mitochondria-UCP1 by uncoupling oxidative phosphorylation from adenosine triphosphate (ATP) synthesis [5]. The electrochemical gradient of protons across the inner membrane is produced by the respiratory chain complexes, while UCP1 passively transfers the protons, causing energy expenditure and heat generation [1].

Brown fat is innervated by the sympathetic nervous system and manifests various β adrenergic receptors, with β3 receptors being the most prevalent [6]. Norepinephrine (NE), which is released from sympathetic nerves, causes triglyceride disruption and activates BAT-UCP1 through the resulting free fatty acids [7].

Performing as an endocrine tissue, BAT secretes a variety of metabolism-enhancing adipokines (BATokines) including Fibroblast growth factor 21 (FGF21), Interleukin 6 (IL6), Growth differentiation factor (GDF15), Bone morphogenetic protein 8b (Bmp8b), Angiopoietin-like 8 (ANGPTL8), Neuregulin 4 (NRG4), Slit guidance ligand 2 (SLIT2), Ependymin related 1 (EPDR1), and Phospholipid transfer protein (PLTP) [8,9,10,11].

In addition to its high energy consumption BAT is considered to be an important target to prevent obesity and metabolic disorders [12,13]. This theory is supported by reports that activated BAT significantly increases whole-body energy expenditure by oxidizing substantial amounts of glucose and fats [13]. Hence, BAT activity is inversely correlated with obesity and insulin resistance [14,15,16]. At the same time, people with active BAT are said to have better insulin sensitivity and a lower incidence of diabetes [17]. Moreover, the presence of triglycerides in BAT is linked with decreased insulin sensitivity, highlighting the importance of BAT in type II diabetes [17].

Although brown fat is considered beneficial in diabetes and obesity prevention, it can also have a negative impact on patients. BAT has been previously linked with many malignancies [1] and was found to contribute to the development of complications such as Cancer-induced Cachexia (CiC) [8]. Moreover, research has shown that some diseases can influence BAT activity [18,19]. Such is the case of patients with CiC where the production of IL-6 and tumour-derived parathyroid hormone-related protein (PTHrP) activates BAT and BeigeAT, which leads to an increased energy consumption [17]. Therefore, it is becoming more widely accepted that inhibiting BAT hyperactivity might be an efficient way to reverse CiC.

**Figure 1:**
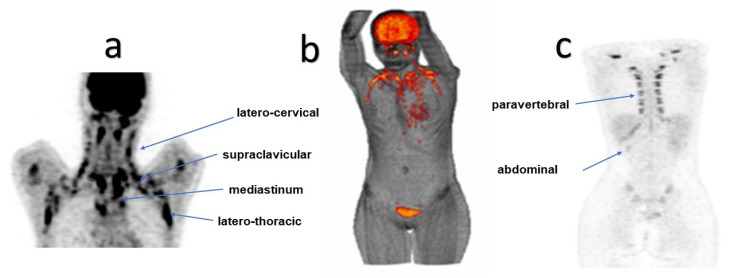
Spectacular BAT activation in the most common BAT localisations was shown in these ^18^F-FDG PET coronal images (**a**,**c**), as well as in ^18^F-FDG PET/CT 3D reconstruction (**b**), in one of our patients.

BAT was initially found in small hibernating mammals, as a thermogenic fat tissue [8]. However, the introduction of fluorine 18 (^18^F) fluorodeoxyglucose (FDG) positron emission tomography (PET)/computed tomography (CT) in clinical oncology practice and the discovery of very high glucose uptake in the cervical and supraclavicular regions led to the incidental discovery of BAT in adults [3,12,14,20]. The Brown Adipose Reporting Criteria in Imaging STudies (BARCIST) criteria, which were recently introduced for a standardised acquisition and evaluation of PET images in this condition, improve the reproducibility of BAT imaging studies. The ^18^F-FDG PET/CT remains the gold standard for BAT identification [21]. Thus, active BAT was predominantly found in the latero-cervical and supraclavicular areas, but other common locations include the mediastinal, paravertebral, latero-thoracic, abdominal, and perirenal regions [22,23,24] (Figure 1).

Given the fact that a variety of factors can affect the ^18^F-FDG uptake in BAT, such as fasting status, diabetes, muscle activity, some medications such as beta-blockers, along with various patient-specific demographic and anthropometric factors, such as age, sex, and body mass index (BMI), it is required to take into consideration these factors prior to the examination [3].

There are several unknowns regarding the physiology of brown fat activity in adults, including the precise quantity of inactive but possibly recruitable BAT. Since BAT makes up a relatively small part of the total human tissue, its true effects on obesity and metabolic illnesses are still being debated [25,26].

Considering all BAT features, this type of fat represents a potential tool in obesity-related research and a possible explanation for CiC. To better understand and enhance our knowledge regarding BAT physiology, we aim to study the ^18^F-FDG BAT biodistribution in a group of oncological patients and look for possible correlations between BAT activity and different primary diseases as well as patients’ weight. We believe that clearing up the unknown about BAT will bring a better understanding of the body’s metabolism regulation system and pave the way for improving patient outcomes.

## 2. Materials and Methods

### 2.1. Patients

The cohort comprised patients referred to the Nuclear Medicine Laboratory of the Regional Institute of Oncology, Iasi, Romania, between 2017 and 2021 for various oncological diagnoses, including Cervical Cancer, Hodgkin’s Lymphoma, Non-Hodgkin’s Lymphoma, Breast Cancer, Lung Cancer, and Gastrointestinal cancers.

**Figure 2:**
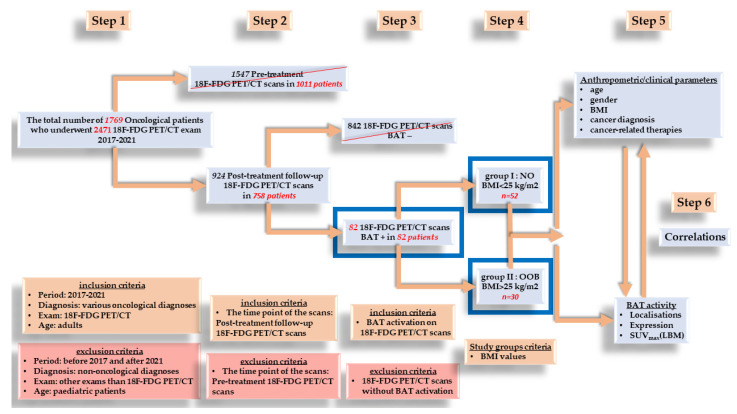
Schematic representation of the research design.

Considering the inclusion and exclusion criteria (exposed in Figure 2), out of the total number of 1769 patients, only 82 patients presented active BAT on the performed ^18^F-FDG PET/CT scans, thus being introduced in the study group.

The patients with active brown fat selected for the study were all at similar stages of disease progression and the selected scans were performed for post-treatment follow-up (at least 2–4 weeks after the last cycle of chemotherapy/2–3 months after radiotherapy/6 weeks after surgery, to prevent possible treatment effects, such as inflammation/infection, on ^18^F-FDG biodistribution), following the national and European guidelines.

We evaluated the patients’ demographic and anthropometric factors related to ^18^F-FDG BAT activity, such as age, gender, BMI, time of year when PET/CT scans were performed, use of medications acting on the beta-adrenergic receptors (beta-blockers/agonists), cancer diagnosis, and cancer-related therapies (chemotherapy, radiotherapy, surgical treatment), and assessed their correlation with brown fat expression.

Based on their BMI, the patients were split into two groups: group I, nonobese (NO), with BMI less than 25 kg/m^2^; group II, overweight and obese (OOB), with BMI greater than 25 kg/m^2^ (Figure 2).

The institutional guidelines were followed during each step of the examination. Knowing that The Nuclear Medicine Laboratory is part of a university hospital, before every examination, the patients provided informed consent for the potential use of their medical data for research. Being retrospective and anonymous, no additional ethical approval was required for this investigation.

### 2.2. ^18^F-FDG PET/CT Scanning Protocol

All the procedures were completed in accordance with the European Association of Nuclear Medicine (EANM) practice guidelines for ^18^F-FDG PET/CT in tumour imaging [27].

Prior to the scan, the patients fasted for at least 6 h. The patients stayed in our laboratory under thermo-neutral conditions (22–24 °C) during the entire procedure so that the outside temperature did not impact BAT expression.

Before the administration of ^18^F-FDG, their blood glucose levels were measured, ranging between 63 and 115 mg/dL. The administered dose of ^18^F-FDG iv-mean was 336,67 MBq (dose interval: 124–544 MBq). To reduce the radiotracer uptake in muscles, the patients remained sat or supine and silent during the injection and the ensuing uptake phase. The imaging procedure started approximately 1 h after ^18^F-FDG administration.

In order to prevent beam-hardening artefacts in the abdomen and pelvis as well as artefacts brought on by truncating the measured Field of view (FOV), the patients were typically positioned with the arms elevated and supported above the head. The scan had a good exposure of the area between the base of the skull and mid-thigh, which is sufficient for the majority of oncological pathologies. Patients with tumours that have a high likelihood of distant metastases to the head, skull, brain, and lower extremities (like in the case of melanoma) underwent extensive whole-body scans.

All the scans were performed with GE Discovery 710 16 Slice PET/CT. The scanning protocol included a scanogram/scout scan/topogram and a low-dose CT scan for attenuation correction (CT-AC) and anatomical correlation followed by PET acquisition. Depending on the reason for performing the CT scans, certain acquisition parameters were selected such as tube current, voltage, slice thickness, rotation time, and pitch. PET data were acquired with an acquisition time of 2 min/bed, 8-bed positions.

### 2.3. Image Processing and Interpretation

All the image processing and interpretation procedures were based on BARCIST criteria [21]. 

After analysing the total number of 2471 oncological ^18^F-FDG PET/CT scans (some patients underwent more than one ^18^F-FDG PET/CT exam for assessing the treatment efficiency), two nuclear medicine physicians reported the presence or absence of BAT in the latero-cervical, supraclavicular, paravertebral, axillary, mediastinal, and abdominal regions [21]. This process was completed by taking into account the characteristic distribution of brown fat, as well as the areas of physiological/pathological ^18^F-FDG uptake. A third nuclear medicine doctor was consulted to settle a potential disagreement.

To accurately study and identify the active BAT pattern, we drew a Region of Interest (ROI) and measured the SUV_max_ normalised to lean body mass (LBM) in every active BAT localisation.

A set of criteria was implemented to ensure bias is reduced and precision improved in the analysis. Thus, a lower cut-off SUV_max_(LBM) value of 1.2 g/mL was set for defining active BAT in areas where the density corresponded to fat tissue in CT imaging (Hounsfield Units −10 to −190) [21]. Moreover, the ROI was drawn at a sufficient distance from visible lymph nodes which represent potential findings in lymphoma patients [12,21].

Regions with SUV_max_(LBM) below the cut-off value were not taken into consideration as they do not represent BAT activity [21].

The measured SUV_max_(LBM) values have been used in order to identify potential correlations between BAT biodistribution and primary diagnoses as well as patients’ demographic and anthropometric parameters.

### 2.4. Statistical Analysis

The statistical data analysis was performed using STATA 16 software (StataCorp LLC, 4905 Lakeway Drive, College Station, TX 77845-4512, USA) and SPSS v.29 (IBM Ireland Product Distribution Limited, IBM House, Shelbourne Road, Ballsbridge, Dublin 4, Ireland). The continuous variables were presented as mean (deviation standard) or median (interquartile range). The comparison tests applied for the continuous numerical variables were selected based on the distribution of the series values and the number of cases included in the analysis. Thus, the Mann–Whitney U Test or Student’s *t*-test was applied for the continuous numerical variables. The Levene test was used to assess the homogeneity of variances. The Kolmogorov–Smirnov test was applied to verify the normal distribution of the variables. For qualitative variables, we analysed frequencies (absolute—n and relative—%) and performed comparisons between the groups based on the results of the non-parametric Pearson Chi-square test. The univariate correlation analysis was completed based on the results of the Pearson correlation test. The multivariate analysis of prognostic factor values was achieved using a multiple linear regression model. The threshold for statistical significance was set at *p* < 0.05.

## 3. Results

Out of the total number of 82 patients who presented active BAT (4.63% of all patients), 52 (63.4%) were nonobese (NO), while 30 (36.6%) were overweight and obese (OOB) (Figure 2) (Table 1).

The mean value (mv) of patients’ ages was 33.8 ± 12.5 years (group I: NO; 32.8 ± 11.8 years vs. group II: OOB; 35.4 ± 13.7 years). Females presented a higher proportion of active BAT (58.5%) than males (41.5%), with no significant differences between genders in the BMI groups (*p* = 0.837). We noticed that 65.9% of cases were reported in cold seasons (autumn and winter), with a significant predominance of BAT expression in NO during spring, summer, and autumn when compared to winter in OOB. The BMI mv was 24.75 ± 5.02 kg/m^2^ (NO: 21.90 ± 2.29 vs. OOB: 29.69 ± 4.62) and presented an important difference between the groups (*p* < 0.001). The blood glucose levels were within normal limits (mv = 90.83 ± 10.93 mg/dL; NO: 91.58 ± 10.89 vs. OOB: 89.53 ± 11.07) for all patients. None of our cases was diagnosed with diabetes.

Our data showed that 31.7% of patients were diagnosed with Hodgkin’s Lymphoma (HL), 14.6% with Non-Hodgkin’s Lymphoma (NHL), 14.6% with Lung Cancer (LC), 13.4% with Cervical Cancer (CC), 12.2% with Breast Cancer (BC), and 13.4% with Gastrointestinal cancers (GCs). We noticed the predominance of each of these diagnoses in NO.

A total of 70.7% of patients were treated surgically, out of which 48.8% also had adjuvant chemotherapy and 19.5% had both radiotherapy and chemotherapy alongside surgical resections. A significant difference was found in the case of NO treated non-surgically but with radiotherapy or with the combination of radiotherapy and chemotherapy, while in OOB patients we noticed a high frequency of cases treated surgically without any adjuvant therapy (chemotherapy or radiotherapy).

Regarding brown fat pattern and quantification (Table 2), ^18^F-FDG high uptake in BAT was described in a unique localisation in only 10 cases (12.2%), while the rest of the patients presented multiple active spots, without a significant difference between NO and OOB (*p* = 0.245).

Brown fat was non-homogeneous in 56.1% of the scans with a greater prevalence in OOB (60%) in comparison with NO (53.9%), where BAT was more homogeneous (NO: 46.2% vs. OOB: 40%). BAT showed a symmetric distribution in 70.7% of patients and 73.1% in NO vs. 66.7% in OOB.

SUV_max_(LBM) in functional BAT ranged between 1.45 and 21.76 g/mL, with the highest mvs in OOB patients of 6.37 ± 3.40 g/mL in supraclavicular, 4.50 ± 2.51 g/mL in latero-cervical (significant difference between the groups; *p* = 0.026) and 3.26 ± 4.07 g/mL in paravertebral spots; however, greater SUV_max_(LBM) mvs of 2.52 ± 4.40 g/mL in mediastinal and 2.27 ± 3.24 g/mL in latero-thoracic (significant difference between the groups; *p* = 0.014) regions were detected in the NO group. Moreover, no one from the OOB group presented abdominal BAT expression.

**Figure 3:**
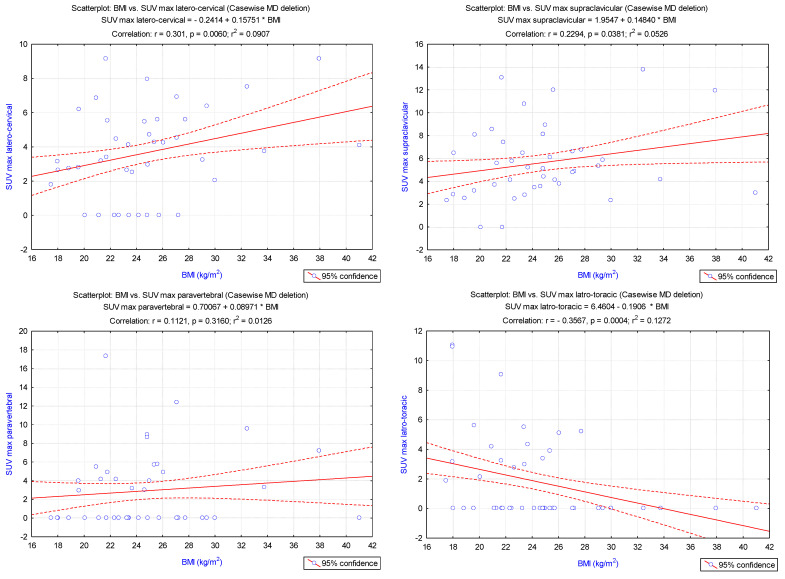

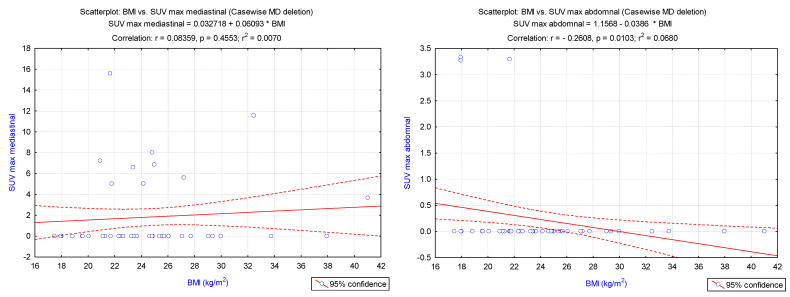
Pearson correlations between the BMI and SUV_max_(LBM) in latero-cervical, supraclavicular, paravertebral, latero-thoracic, mediastinal, and abdominal localisations.

BMI was positively and significantly correlated with latero-cervical (*p* = 0.006) and supraclavicular (*p* = 0.038) SUV_max_(LBM), while this index showed a negative correlation with latero-thoracic (*p* = 0.0004) and abdominal (*p* = 0.01) SUV_max_(LBM). Furthermore, SUV_max_(LBM) in paravertebral (*p* = 0.3) and mediastinal (*p* = 0.08) spots presented no correlations with BMI values (Figure 3). 

**Figure 4:**
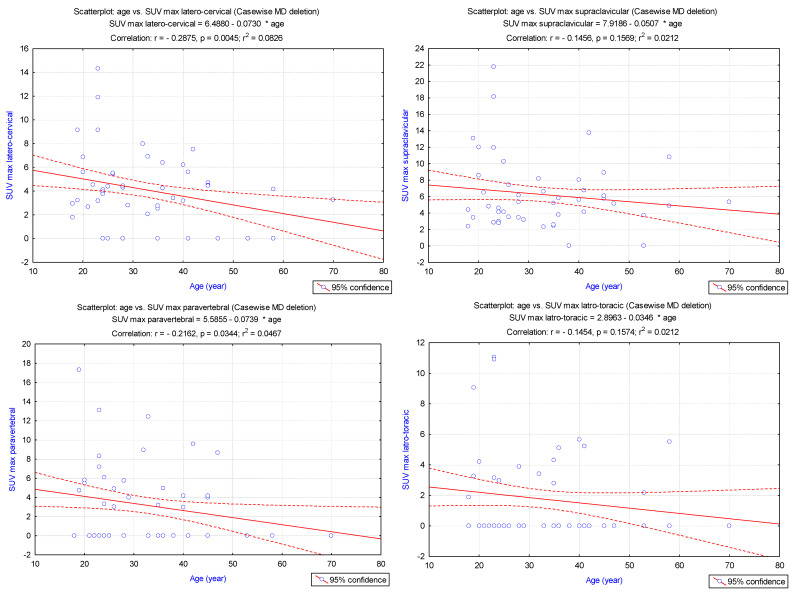

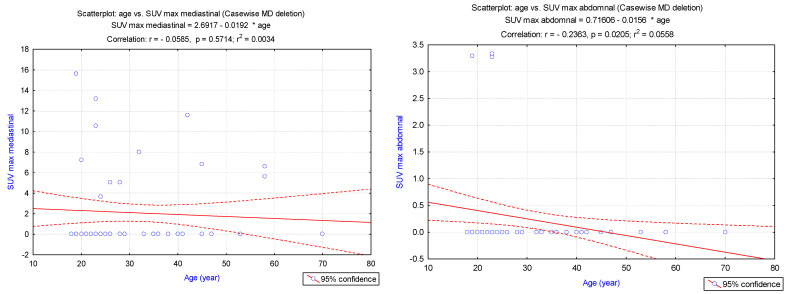
Pearson correlations between the age and SUV_max_(LBM) in latero-cervical, supraclavicular, paravertebral, latero-thoracic, mediastinal, and abdominal localisations.

Considering the age of the patients, SUV_max_(LBM) decreases significantly in latero-cervical (*p* = 0.004), paravertebral (*p* = 0.034), and abdominal (*p* = 0.02) spots. Nevertheless, we noticed that the supraclavicular (*p* = 0.16), latero-thoracic (0.16), and mediastinal (*p* = 0.57) SUV_max_(LBM) expressed no important correlations with age (Figure 4). 

**Figure 5:**
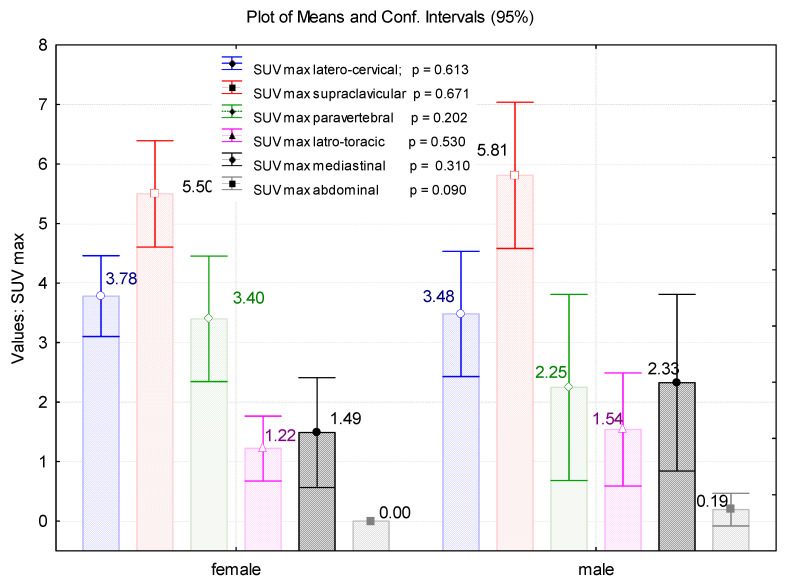
Evaluation of the SUV_max_(LBM) values in latero-cervical, supraclavicular, paravertebral, latero-thoracic, mediastinal, and abdominal localisations, depending on the gender of the patients.

By measuring the paravertebral SUV_max_(LBM), we demonstrated that men had significantly lower BAT activity in this region than women in comparison with the mediastinal localisation, which had higher SUV_max_(LBM) values in males. After all, the BAT expression in latero-cervical, supraclavicular, latero-thoracic, and abdominal localisations represented no important differences between the gender groups (Figure 5).

**Figure 6:**
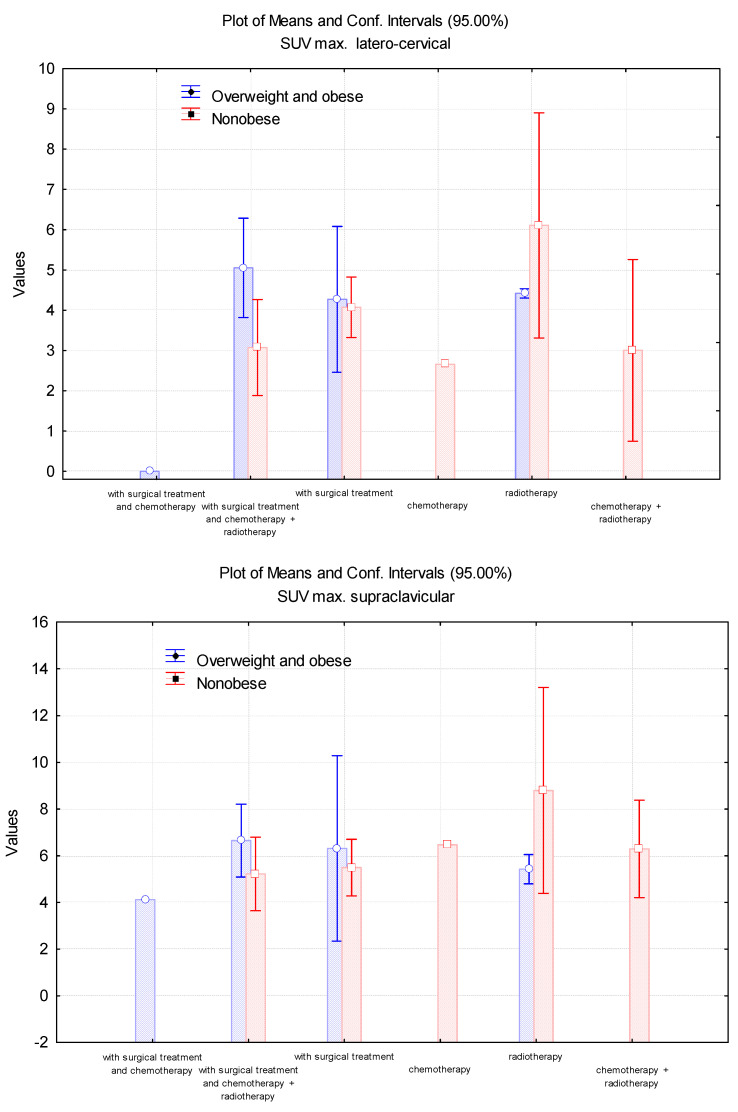

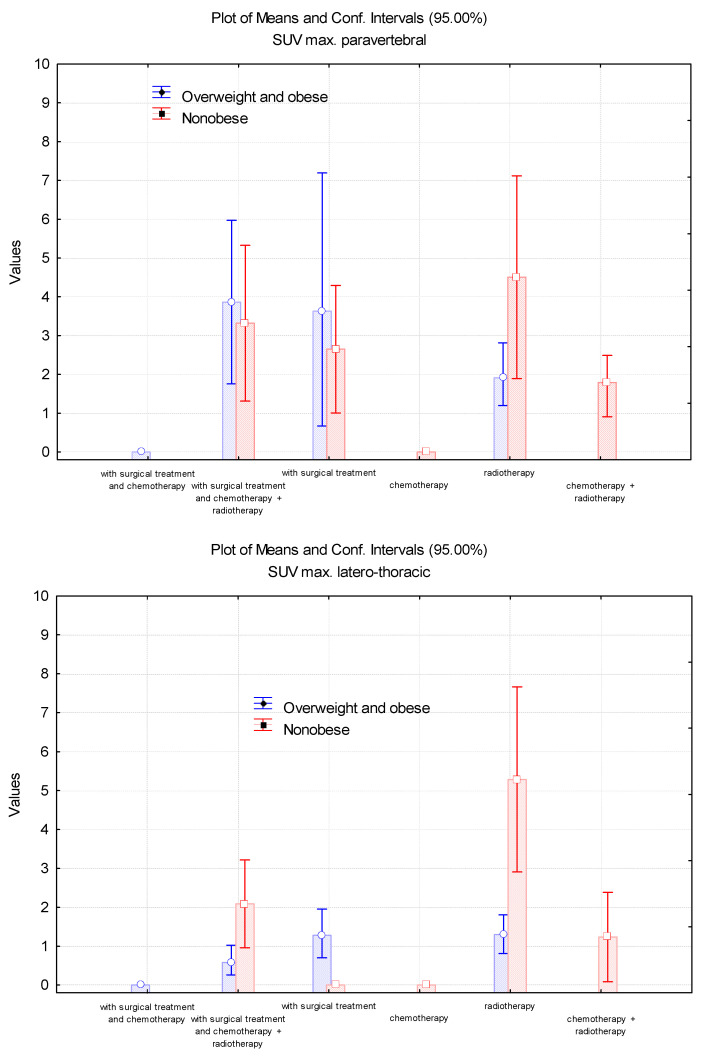

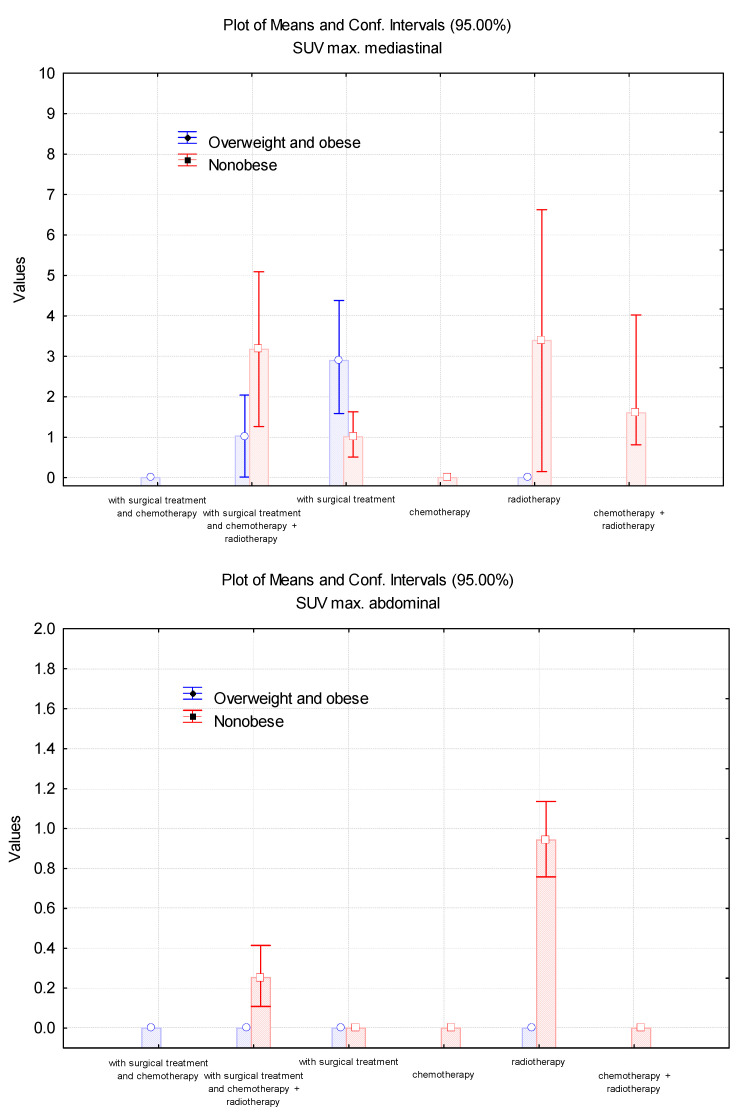
Evaluation of the SUV_max_(LBM) values in latero-cervical, supraclavicular, paravertebral, latero-thoracic, mediastinal, and abdominal localisations, depending on the treatment strategies.

The highest values of SUV_max_(LBM) in all BAT localisations were noted in NO patients treated only with radiotherapy. Nevertheless, BAT expression was greater in OOB patients who had only surgical treatment. The lowest values of SUV_max_(LBM) were identified for NO people treated with only chemotherapy. Considering the mediastinal, latero-thoracic, and abdominal SUV_max_(LBM), we showed that NO patients treated surgically in addition to the combination of radiotherapy and chemotherapy had greater values than OOB patients who followed the same treatment strategy, contrary to latero-cervical, supraclavicular, and paravertebral SUV_max_(LBM), which were higher among the OOB group (Figure 6).

**Figure 7:**
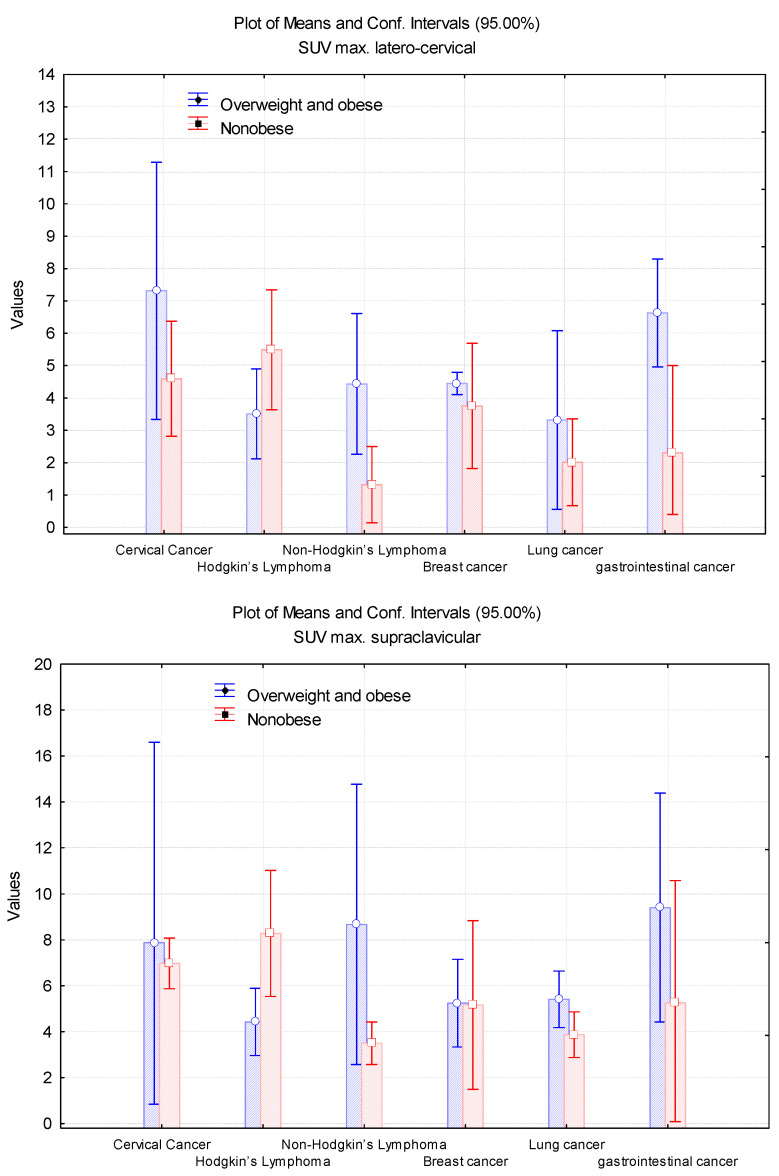

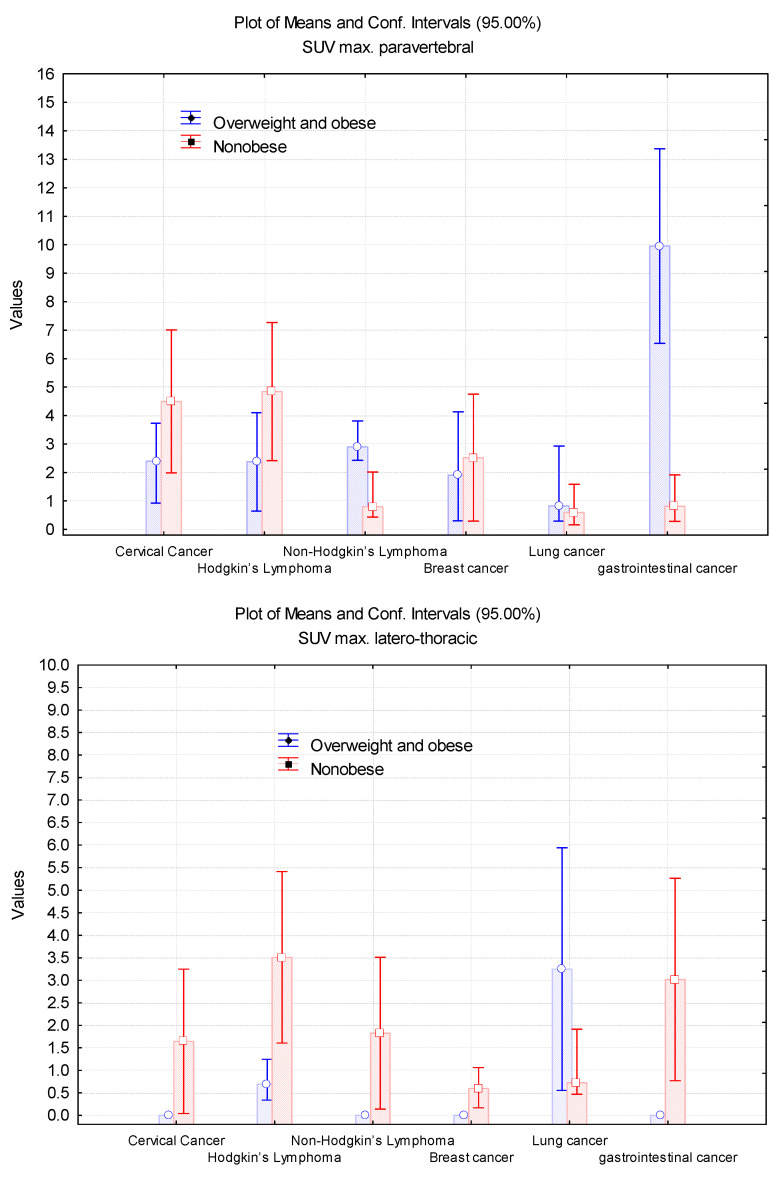

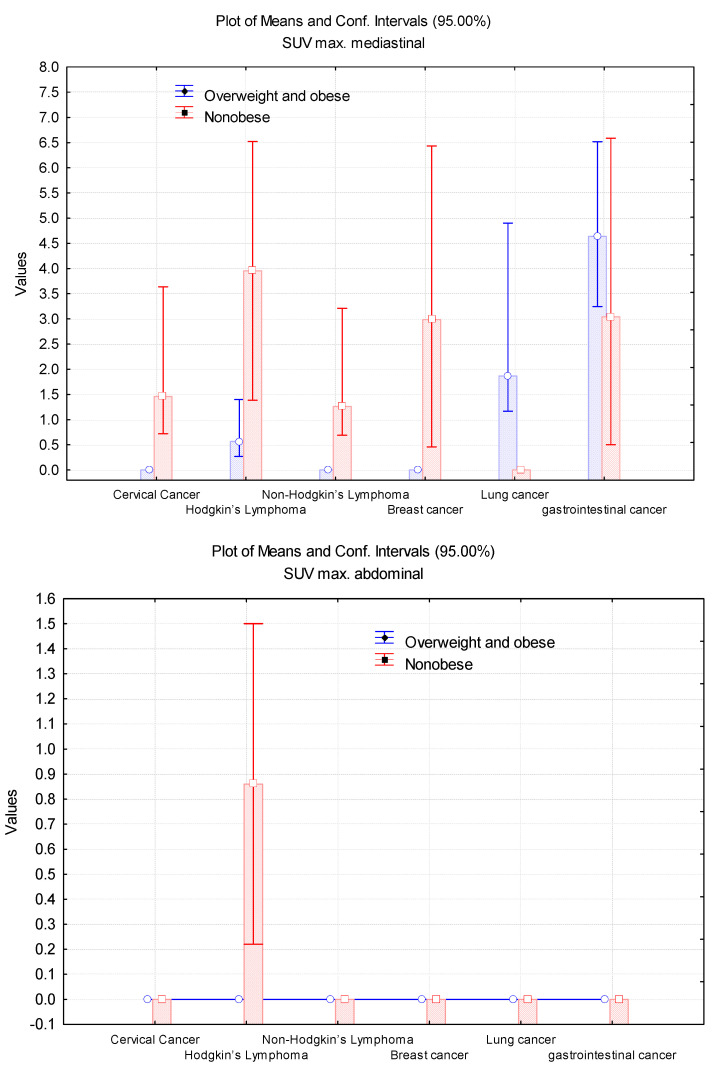
Evaluation of the SUV_max_(LBM) values in latero-cervical, supraclavicular, paravertebral, latero-thoracic, mediastinal, and abdominal localisations, depending on the diagnosis.

OOB individuals with CC had the highest SUV_max_(LBM) values in latero-cervical and supraclavicular spots compared to OOB patients with GCs in the paravertebral area and OOB patients with LC in the latero-thoracic region. NO patients with HL presented the highest brown fat expression in all BAT main areas; thus, the OOB group with LC had the greatest SUV_max_(LBM) values in all BAT spots. In comparison with the NO group, OOB patients showed more important BAT activity in cases with CC (in latero-cervical and supraclavicular regions), NHL (in latero-cervical, supraclavicular and paravertebral regions), BC (in latero-cervical region), and LC (in all BAT regions) (Figure 7).

To summarize, our resulting correlations showed that BMI significantly influences the latero-cervical, latero-thoracic, and abdominal SUV_max_(LBM). It was also noted that age has an important impact on the vast majority of SUV_max_(LBM) values, specifically in the latero-cervical, paravertebral, and abdominal localisations. In addition, it was shown that gender can influence SUV_max_(LBM) values (the male patients presented significantly lower measurements).

The supraclavicular and mediastinal SUV_max_(LBM) values were not affected by any of the five parameters that we analysed in Table 3: age, gender, BMI, treatment strategy, and diagnosis.

## 4. Discussion

Following the “rediscovery” of the BAT presence in human adults in 2009 [4,14,28], this type of fat has been anticipated as a great therapeutic target for the treatment of obesity. Yet, when triggered improperly, BAT can also have negative outcomes.

It was hypothesised as early as 1981 that BAT activation induces a hypermetabolic state and contributes to weight loss in cancer patients [29], which was contrary to the previously widely held belief that BAT is only present in newborns and vanishes in adults.

In accordance with this hypothesis, we noticed in our results a significant correlation between BMI and BAT activity, with an important difference in the distribution of brown fat among NO vs. OOB. These findings emphasise the potential implication of BAT activity in the oncological patient’s weight status. In addition, the fact that BMI was positively and significantly correlated with latero-cervical and supraclavicular SUV_max_(LBM), while this index showed a negative correlation with latero-thoracic and abdominal SUV_max_(LBM), demonstrates that the localisation, as well as the amount of activated BAT, could influence this fat’s effects on BMI. 

The impact of BAT expression on our oncological patient’s weight status, in addition to the BMI mv, which was 24.75 ± 5.02 kg/m^2^ (NO: 21.90 ± 2.29 kg/m^2^), makes us consider the hypothesis of BAT implication in CiC occurrence.

The most recent definition of CiC includes weight loss of more than 5% over the last six months or more than 2% in cases who already exhibit a lower weight status (BMI < 20 kg/m^2^) or skeletal muscle index (sarcopenia) [30]. This particular form of cachexia is acknowledged as a separate clinical disease that has an adverse effect on prognosis, therapeutic success, and quality of life.

**Figure 8:**
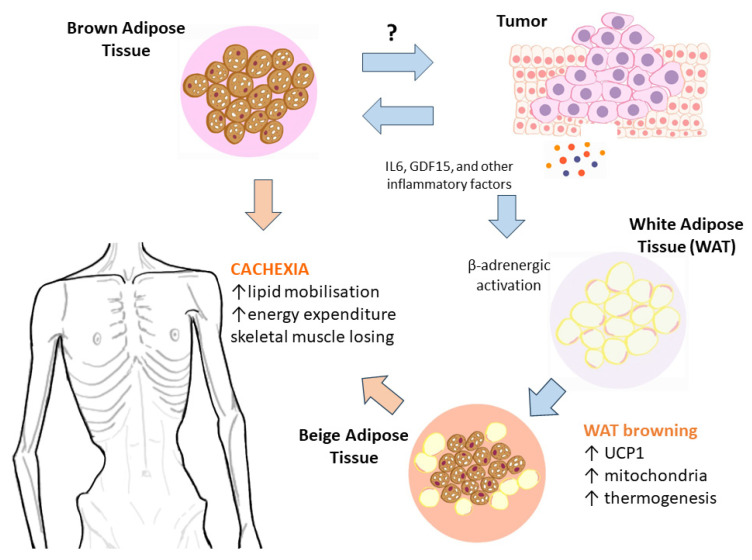
Schematic representation of the possible BAT/BeigeAT implication in CiC production.

It was previously claimed that BAT could have contributed to the development of CiC through its BATokines, IL6, and GDF15, which work simultaneously in an autocrine/paracrine manner [8,9,31]. Circulating GDF15 and IL6, derived from cancer cells, could perhaps activate brown fats [8]. When these mediators’ values exceed a certain threshold, a positive feedback cycle of inter-BAT mutual activations would be produced to cause uncontrolled catabolism as well as CiC [8] (Figure 8).

In our work, NO patients with HL presented the highest brown fat expression in all BAT main localisations; however, BAT activity in NO cases with gastrointestinal cancer was lower than the OOB group with the same diagnosis. Previous studies indicated that CiC occurs more commonly in individuals with lung and upper gastrointestinal cancers and less frequently in those with breast cancer or lower gastrointestinal cancer [30]. These results, besides our findings, support the hypothesis which indicates that the development of CiC may depend on the type of oncological diagnosis.

As an explanation for this supposition, recent evidence suggests that cachexia is caused by a central perturbation of the hypothalamic pathways controlling energy homeostasis, which leads to anorexia and decreased energy intake, increased energy expenditure, and loss of body tissue, such as muscle proteolysis and lipolysis [30].

Up to 70% of total energy expenditure in sedentary adults is made up of resting metabolic rate (RMR) [30]. Certain cancer patients have been reported to express an increased hypermetabolism, which was identified in 50% of patients with mixed tumours and up to 74% of individuals with primary lung cancer [30]. RMR is higher in cancer patients who lose weight [30]. There are several pathophysiological explanations for an abnormal elevation of RMR in CiC, including abnormalities in the carbohydrates, fats, or proteins metabolism, systemic inflammation, hyperadrenergic activity, and an enlarged liver mass [30].

Taking into account that 31.7% of our patients were diagnosed with HL (important predominance compared with the other diagnoses), along with the fact that there was an important activation of brown fat identified in these patients (high SUV_max_(LBM) values), we may consider that BAT could potentially play a role in the development of cancer itself. Preceding research reported that breast cancer has been associated with an increase in a variety of circulating BATokines [32]. These adipokines stimulate many pathways that aid in the development and hallmarks of this type of malignancy [32].

In a study where human breast cancer cells were transplanted to a xenotransplantation mice model, both the host microenvironmental cells and transplanted grafts showed early brown adipose (BA)-selective gene inductions [8,31]. The acquisition of BA phenotypes is not a consequence but rather a cause of cancer progression. This fact was evidenced by the considerable reduction in tumour development following the elimination of BA-tilted cells that were positive for UCP1 or Myogenic Factor 5 (MYF5) [8]. Due to several similarities between BATs and mammary glands, it is not known whether this phenomenon is unique to breast malignancies or prevalent in other tumours as well.

We already know the following: (1) during postnatal development, BAT appears in mammary glands; (2) brown fat and mammary glands are grouped in BioGPS due to the similarity in their gene expression profile; (3) the formation of BAT and mammary fat pads is not dependent on the *CEBPA* gene [8]. A risk assessment should be carefully carried out when BAT-based anti-obesity therapy development is taken into consideration since evidence linking brown fat to the occurrence of other cancers is still lacking.

Previous research has yielded conflicting conclusions regarding the involvement of cancer vitality in the regulation of BAT activity [33,34]. Except for the fact that males had significantly lower activation of BAT, our results were similar to the findings of Brendle C et al. who showed that apart from age (SUV_max_(LBM) decreases significantly in some BAT regions with the patient’s age, which is comparable with our results), none of the other investigated parameters, such as sex, disease activity, and treatment approach, were connected to brown fat activation in lymphoma patients [18]. Moreover, they demonstrated in a univariate regression that BAT activity was significantly higher in patients with HL in comparison with patients with mature B-cell lymphoma (*p* = 0.004) [12]. These findings supported the predominance of our HL patients with active BAT, in addition to the important activation of brown fat in these patients. On the other hand, Gilsanz et al. proposed that active lymphoma may inhibit BAT activation showing that brown fat expression was lower in their patient group at the time of lymphoma diagnosis than in the follow-up period with inactive disease [35]. Conversely, Brendle C et al. showed in other studies that it is unlikely that lymphoma reduces BAT activity since brown fat has the same expression in patients with other types of malignancies [18], concluding that BAT activity was not shown to be linked to any specific type of lymphoma [12].

In our work, we did not find significant correlations between the oncological diagnosis and SUV_max_(LBM) values. These findings are in line with the previous studies that were unable to confirm whether any primary malignancy had an impact on BAT activity since the connection to any primary disease was not an independent predictor for brown fat expression in all these multivariate analyses studies.

Regarding the effects of cancer-related therapy on brown fat activation, some research, including ours, reported no connection between brown fat expression and different treatment strategies [18]. However, a significant difference was found in our NO patients treated non-surgically but with radiotherapy or with the combination of radiotherapy and chemotherapy, while in OOB patients we noticed a high frequency of cases treated surgically without any adjuvant therapy (chemotherapy or radiotherapy). These results could support the hypothesis that different treatment strategies could have various effects on the expression of BAT among the BMI groups.

It is debatable how prior chemotherapy affects BAT activity [18,36,37]. In our treated patients with chemotherapy, BAT presented a metabolic activity only in latero-cervical (the lowest SUV_max_(LBM) mv) and supraclavicular localisations. Brendle C et al. conducted a study that showed ABVD treatment was linked to a higher prevalence of BAT expression (*p* = 0.01) [12]. However, based on a comprehensive retrospective investigation involving numerous tumours, Steinberg et al. suggested that chemotherapy might reduce BAT [36]. Moreover, other studies demonstrated that brown fat was more present in patients without prior lymphoma-related therapy, confirming the inhibitory effects of chemotherapy or radiotherapy on BAT [12]. Furthermore, chemotherapy has been shown to decrease BAT activity in patients with breast cancer [36]. 

The highest values of SUV_max_(LBM) in all BAT localisations were noted in our group of NO patients treated only with radiotherapy. These results conflict with the case report paper, published by Gnaneswaran S et al. about the abolishment of brown fat FDG uptake by radiotherapy in a woman with non-small-cell lung cancer [38]. Taking into consideration the lack of data in the literature, this discord raises the necessity of further detailed research regarding the possible local/regional/total body radiotherapy effects on BAT expression in oncological patients.

In contrast with all these potential BAT negative effects and implications in oncology, Seki T et al. demonstrated that cold-induced BAT activation in tumour-bearing mice significantly suppresses the development of a variety of solid tumours, including malignities that are currently incurable, like pancreatic cancer [39]. This function could be explained by the fact that this fat considerably lowers blood glucose levels and prevents cancer cells from using a glycolysis-based metabolism [39], which also could illustrate the normal blood glucose levels that were determined in our patients (mv = 90.83 ± 10.93 mg/dL; NO: 91.58 ± 10.89 vs. OOB: 89.53 ± 11.07).

**Figure 9:**
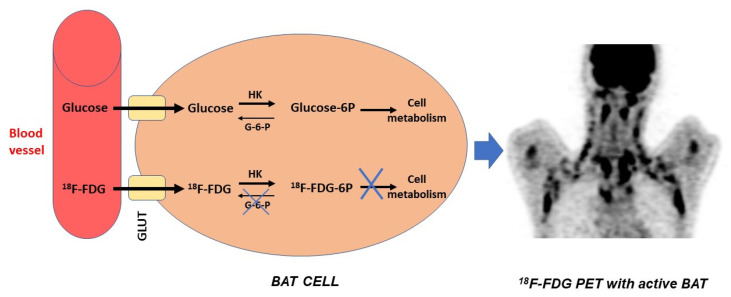
Schematic representation of ^18^F-FDG uptake mechanism in BAT.

Due to a variety of factors, ^18^F-FDG is considered the most often used radiopharmaceutical for imaging BAT and observing its metabolic activity. In tissues with a high glucose turnover, such as the brain, active muscle tissue, and tumours, ^18^F-FDG is characterised by a quick and appropriate biodistribution in addition to an irreversible uptake [40]. This radiotracer traverses the cell membrane with the help of the “glucose transporters family (GLUT)”, more often GLUT1, to be phosphorylated by hexokinase and transformed in ^18^F-FDG-6 phosphate [1]. This new compound is locked inside the cell without being metabolised further [1] (Figure 9).

Afterwards, PET/CT imaging allows physicians to measure and analyse the radiopharmaceutical uptake in the tissue. Knowing that ^18^F-FDG uptake is strongly correlated with the expression of UCP-1 and increased energy expenditure in response to a cold stimulus [1], ^18^F-FDG PET/CT is considered the gold standard for non-invasive BAT detection. Although ^18^F-FDG indirectly measures the tissue’s ability to take up glucose, it does not have the capacity to analyse the BAT’s mitochondrial activity; therefore, other radiopharmaceuticals such as ^99m^Tc-sestamibi (Single-photon emission computed tomography (SPECT) radiotracer) were used for brown fat studies [41,42].

It is crucial to take into account various limitations while analysing the findings of our current study. The fact that we had to include only individuals with active BAT in order to analyse this tissue’s pattern explains the small number of patients included in the study groups. These patients are a minority as it is widely known that BAT activation in adults only occurs under very specific circumstances. Considering this limitation (small samples), specific statistical tests were used to validate the hypotheses. Therefore, the statistical power of the estimates was maintained at an acceptable level in the univariate statistical analysis utilised for comparisons.

Another important aspect is that the odd position and frequent asymmetry of hypermetabolic BAT in the mediastinum and upper abdomen are more prone to be misdiagnosed as primary cancer or nodal metastases. Thus, a thorough analysis of BAT was conducted by our two physicians (with the help of a third one to settle any debates in some cases) based on all available scans and CT images provided.

Even though previous proof-of-concept human studies have clearly shown the value of ^18^F-FDG PET/CT, there are still several significant limitations to this method, including heterogeneity of response, sensitivity to environmental or experimental factors, insensitivity to fatty acid-mediated metabolism (the preferred energy source for BAT), and confounding variables for SUV-based quantitation [3]. Further prospective studies based on specific methodologies and larger samples, as well as developing standardised methods to analyse ^18^F-FDG PET/CT images, would therefore aid the development of more conclusive findings.

## 5. Conclusions

This study emphasises the potential implication of BAT activity in the oncological patient’s weight status and demonstrates that the localisation, along with the amount of activated BAT, could influence this fat’s effects on BMI. These findings support the hypothesis of brown fat’s involvement in CiC occurrence, which may depend on the type of oncological diagnosis.

In contrast with all these potential BAT negative effects and implications in oncology, which have been supported by our results, it was previously reported that brown fat could significantly suppress the development of a variety of solid tumours. Thus, BAT represents a double-edged sword with mixed effects. Therefore, it is mandatory to keep its activity within suitable parameters (localisation/quantity) in order to optimise the clinical management of oncological patients, as well as during the development of therapies based on this type of tissue, especially in the field of personalised medicine in oncology.

## Figures and Tables

**Table 1:** The demographic and clinical characteristics of patients with oncological pathologies and active BAT.

Clinical Characteristics	Total Patients N = 82	Nonobese Patients (NO) (BMI ≤ 25 kg/m^2^) N = 52	Overweight and Obese (OOB) (BMI > 25 kg/m^2^) N = 30	*p*-Value
Age, median (IQR), year Age, mean (SD), year	33 (24–41) 33.8 (12.5)	30.5 (23–40) 32.8 (11.8)	33 (24–41) 35.4 (13.7)	0.375 *
Gender, female/male, n (%)	48/34 (58.5/41.5)	30/22 (62.5/64.7)	18/12 (37.5/35.3)	0.837 ^
**Season, n (%)** spring summer autumn winter	18 (22) 10 (12.2) 30 (36.6) 24 (29.3)	12 (66.7) 6 (60) 24 (80) 10 (41.7)	6 (33.3) 4 (40) 6 (20) 14 (58.3)	0.0335 ^
Body weight, median (IQR), kg Body weight, mean (SD), kg	70 (62–78) 71.5 (15.82)	63 (58–70) 63.5 (8.72)	80 (74–95) 85.3 (15.9)	<0.001 *
BMI, median (IQR), kg/m^2^ BMI, mean (SD), kg/m^2^	24.16 (21.68–27.06) 24.75 (5.02)	22.05 (20.06–23.67) 21.90 (2.29)	27.72 (26.06–32.47) 29.69 (4.62)	<0.001 *
Blood glucose level, median (IQR), (mg/dL) Blood glucose level, mean (SD), (mg/dL)	91 (82–95) 90.83 (10.93)	91 (84–95) 91.58 (10.89)	91 (79–101) 89.53 (11.07)	0.418 *
**Diagnostic, n (%)** Cervical Cancer Hodgkin’s Lymphoma Non-Hodgkin’s Lymphoma Breast Cancer Lung Cancer Gastrointestinal cancers	11 (13.4) 26 (31.7) 12 (14.6) 10 (12.2) 12 (14.6) 11 (13.4)	8 (72.7) 16 (61.5) 8 (66.7) 7 (70) 7 (58.3) 6 (54.5)	3 (27.3) 10 (38.5) 4 (33.3) 3 (30) 5 (41.7) 5 (45.5)	0.952 ^
**Treatment, n(%)** **with surgical treatment** chemotherapy chemotherapy + radiotherapy without chemotherapy/radiotherapy **without surgical treatment** chemotherapy radiotherapy chemotherapy + radiotherapy	**58 (70.7)** 40 (48.8) 16 (19.5) 2 (2.4) **24 (29.3)** 14 (17.1) 2 (2.4) 8 (9.8)	**32 (55.2)** 24 (60) 8 (50) 0 (0) **20 (83.3)** 10 (71.4) 2 (100) 8 (100)	**26 (44.8)** 16 (40) 8 (50) 2 (100) **4 (16.7)** 4 (28.6) 0 (0) 0 (0)	0.012 ^

* *t*-test or Mann–Whitney U Test; ^ Pearson Chi-square.

**Table 2:** BAT pattern and quantification.

	Total Patients N = 82	Nonobese Patients (BMI ≤ 25 kg/m^2^) N = 52	Overweight and Obese Patients (BMI > 25 kg/m^2^) N = 30	*p*-Value
**BAT localisation, n (%)** Unique location Multiple locations	10 (12.2) 72 (87.8)	8 (15.4) 44 (84.6)	2 (6.67) 28 (93.3)	0.245 ^
**BAT, n (%)** homogeneous non-homogeneous	36 (43.9) 46 (56.1)	24 (46.2) 28 (53.9)	12 (40) 18 (60)	0.587 ^
**BAT, n (%)** symmetric asymmetric	58 (70.7) 24 (29.3)	38 (73.1) 14 (26.9)	20 (66.7) 10 (33.3)	0.541 ^
**SUV_max_(LBM) latero-cervical (g/mL)** median (IQR) mean (SD)	3.40(2.06–5.53) 3.66 (2.63)	2.90 (0–4.72) 3.17 (2.59)	4.29 (3.25–6.39) 4.50 (2.51)	0.026 *
**SUV_max_(LBM) supraclavicular (g/mL)** median (IQR) mean (SD)	5.14 (3.45–6.77) 5.63 (3.25)	4.79 (2.84–7.45) 5.20 (3.11)	5.35 (4.12–6.77) 6.37 (3.40)	0.115 *
**SUV_max_(LBM) paravertebral (g/mL)** median (IQR) mean (SD)	0 (0–4.90) 2.90 (4.02)	0 (0–4.16) 2.72 (4.02)	0 (0–5.79) 3.26 (4.07)	0.426 *
**SUV_max_(LBM) latero-thoracic (g/mL)** median (IQR) mean (SD)	0 (0–3.21) 1.76 (2.90)	0 (0–3.40) 2.27 (3.24)	0 (0–5.11) 0.84 (1.85)	0.014 *
**SUV_max_(LBM) mediastinal (g/mL)** median (IQR) mean (SD)	0 (0–1.84) 2.06 (4)	0 (0–5.04) 2.52 (4.40)	0 (0–5.60) 1.23 (3.05)	0.164 *
**SUV_max_(LBM) abdominal (g/mL)** median (IQR) mean (SD)	0 (0–0) 0.21 (0.8)	0 (0–0) 0.32 (0.98)	0 (0–0) 0 (0)	0.434 *

* *t*-test or Mann–Whitney U Test; ^ Pearson Chi-square.

**Table 3:** The coefficients of multiple linear regression regarding the SUV_max_(LBM) and various parameters: age, gender, BMI, treatment strategy, and diagnosis.

Multiple Linear Regression	Unstandardized Coefficients		Standardized Coefficients	*t*	*p*-Value
	B	Std. Error	Beta		
**SUV_max_(LBM) latero-cervical (g/mL)**					
(Constant)	1.532	2.226		0.688	0.493
Age	−0.061	0.024	−0.288	−2.518	0.014
Gender	0.636	0.582	0.120	1.092	0.278
BMI	0.149	0.059	0.285	2.544	0.013 *
Treatment strategy	−0.135	0.212	−0.076	−0.636	0.526
Diagnostic	−0.034	0.175	−0.022	−0.196	0.845
Model verification: ANOVA: F = 3.181, *p* = 0.012, (*) Marked effects are significant at *p* < 0.05.					
**SUV_max_(LBM) supraclavicular (g/mL)**					
(Constant)	3.138	2.936		1.069	0.289
Age	−0.019	0.032	−0.074	−0.608	0.545
Gender	−0.113	0.767	−0.017	−0.148	0.883
BMI	0.141	0.077	0.217	1.817	0.073
Treatment strategy	−0.099	0.279	−0.045	−0.355	0.724
Diagnostic	0.046	0.231	0.024	0.200	0.842
Model verification: ANOVA: F = 0.958, *p* = 0.449, (*) Marked effects are significant at *p* < 0.05.					
**SUV_max_(LBM) paravertebral (g/mL)**					
(Constant)	5.297	3.249		1.630	0.107
Age	−0.100	0.037	−0.292	−2.675	0.009 *
Gender	2.111	0.881	0.250	2.396	0.019 *
BMI	−0.016	0.083	−0.021	−0.195	0.846
Treatment strategy	−0.510	0.317	−0.186	−1.609	0.111
Diagnostic	−0.065	0.267	−0.025	−0.242	0.809
Model verification: ANOVA: F = 2.410, *p* = 0.042, (*) Marked effects are significant at *p* < 0.05.					
**SUV_max_(LBM) latero-thoracic (g/mL)**					
(Constant)	6.417	2.217		2.895	0.005 *
Age	−0.030	0.025	−0.125	−1.170	0.245
Gender	0.380	0.601	0.064	0.632	0.529
BMI	−0.180	0.057	−0.337	−3.178	0.002 *
Treatment strategy	0.090	0.216	0.047	0.418	0.677
Diagnostic	−0.049	0.182	−0.027	−0.268	0.789
Model verification: ANOVA: F = 3.286, *p* = 0.009, (*) Marked effects are significant at *p* < 0.05.					
**SUV_max_(LBM) mediastinal (g/mL)**					
(Constant)	4.962	3.299		1.504	0.136
Age	−0.031	0.038	−0.095	−0.830	0.409
Gender	0.240	0.895	0.029	0.268	0.789
BMI	−0.081	0.084	−0.110	−0.967	0.336
Treatment strategy	−0.204	0.322	−0.077	−0.634	0.528
Diagnostic	0.136	0.271	0.055	0.501	0.618
Model verification: ANOVA: F = 0.338, *p* = 0.888, (*) Marked effects are significant at *p* < 0.05.					
**SUV_max_(LBM) abdominal (g/mL)**					
(Constant)	1.873	0.618		3.029	0.003 *
Age	−0.015	0.007	−0.221	−2.057	0.043
Gender	0.103	0.168	0.063	0.615	0.540
BMI	−0.042	0.016	−0.287	−2.690	0.009 *
Treatment strategy	−0.037	0.060	−0.070	−0.616	0.539
Diagnostic	−0.061	0.051	−0.124	−1.201	0.233
Model verification: ANOVA: F = 2.956, *p* = 0.016, (*) Marked effects are significant at *p* < 0.05.					

## Data Availability

The data presented in this study are available on request from the corresponding author.

## Abbreviations

Brown Adipose Tissue **(BAT)**, fluorine 18 **(18F)**, fluorodeoxyglucose **(FDG)**, positron emission tomography **(PET)**/computed tomography **(CT)**, **(18F-FDG PET/CT)**, nonobese **(NO)**, overweight and obese **(OOB)**, standardized uptake value **(SUV)**, lean body mass **(LBM)**, Body Mass Index **(BMI)**, white adipose tissue **(WAT)**, beige or brite adipose tissue **(BeigeAT)**, uncoupling protein 1 **(UCP1)**, adenosine triphosphate **(ATP)**, BAT adipokines **(BATokines)**, Fibroblast growth factor 21 **(FGF21)**, Interleukin 6 **(IL6)**, Growth differentiation factor **(GDF15)**, Bone morphogenetic protein 8b **(Bmp8b)**, Angiopoietin-like 8 **(ANGPTL8)**, Neuregulin 4 **(NRG4)**, Slit guidance ligand 2 **(SLIT2)**, Ependymin related 1 **(EPDR1)**, and Phospholipid transfer protein **(PLTP)**, Cancer-induced Cachexia **(CiC)**, Brown Adipose Reporting Criteria in Imaging STudies **(BARCIST)**, European Association of Nuclear Medicine **(EANM)**, Field of view **(FOV)**, Region of Interest **(ROI)**, mean value **(mv)**, Hodgkin’s Lymphoma **(HL)**, Non-Hodgkin’s Lymphoma **(NHL)**, Lung Cancer **(LC)**, Cervical Cancer **(CC)**, Breast Cancer **(BC)**, Gastrointestinal cancers **(GCs)**, resting metabolic rate **(RMR)**, brown adipose **(BA)**, Myogenic Factor 5 **(MYF5)**, glucose transporters **(GLUT)**.
